# Plasmonic molecules via glass annealing in hydrogen

**DOI:** 10.1186/1556-276X-9-606

**Published:** 2014-11-08

**Authors:** Alexey Redkov, Semen Chervinskii, Alexander Baklanov, Igor Reduto, Valentina Zhurikhina, Andrey Lipovskii

**Affiliations:** 1 Institute of Physics, Nanotechnology and Telecommunications, St. Petersburg State Polytechnic University, 29 Polytechnicheskaya, St. Petersburg 195251, Russia; 2 Department of Physics and Technology of Nanostructures, St. Petersburg Academic University, 8/3 Khlopina, St. Petersburg 194021, Russia; 3 Institute of Photonics, University of Eastern Finland, Yliopistokatu 7, P.O. Box 111, Joensuu 80101, Finland; 4 Ioffe Physical-Technical Institute of RAS, 26 Polytechnicheskaya, St. Petersburg 194021, Russia

**Keywords:** Plasmonic molecule, Nanoparticle, Nanoisland, Film, Self-assembly, Nanostructuring

## Abstract

Growth of self-assembled metal nanoislands on the surface of silver ion-exchanged glasses via their thermal processing in hydrogen followed by out-diffusion of neutral silver is studied. The combination of thermal poling of the ion-exchanged glass with structured electrode and silver out-diffusion was used for simple formation of separated groups of several metal nanoislands presenting plasmonic molecules. The kinetics of nanoisland formation and temporal evolution of their size distribution on the surface of poled and unpoled glass are modeled.

**PACS:**

78.67.Sc (nanoaggregates; nanocomposites); 81.16.Dn (self-assembly); 68.35.bj (surface structure of glasses); 64.60.Qb (Nucleation); 81.16.Nd (micro- and nanolithography)

## Background

By now, wide-scale studies of glasses embedded with metal nanoparticles have been performed [1-4]. These media are of interest because of surface plasmon resonance in the nanoparticles excited at optical frequencies, which results in the electric field enhancement in the vicinity of the nanoparticles. The latter gives rise to optical nonlinearity, efficiency of Raman scattering, and luminescence of the glasses. An effective technique being used for the formation of metal nanoparticles in glasses is annealing of a glass containing noble metal ions in hydrogen [5]. It has been recently found that instead of or in parallel with the formation of metal nanoparticles in the bulk under hydrogen processing, the growth of metal nanoislands at the surface takes place in certain types of glasses. Nanoisland growth that is formation of metal island film (MIF) on the surface occurs [6] because of out-diffusion of neutral metal from these glasses. In particular, thermal treatment of Ag^+^-rich phosphate [6] and borate [7] glasses in hydrogen results in MIF formation on their surface, whereas growth of nanoparticles in the bulk of the glasses is suppressed. Phosphate and silicate glasses with MIF formed via the out-diffusion have recently been presented as substrates for surface-enhanced Raman spectroscopy (SERS) measurements [6,8]. Metal nanoparticles on the surface are also of interest because being closely placed in a small group they arrange the so-called plasmonic molecules (PM) [9], which demonstrate unique resonant optical properties. Various lithographic techniques and organic templates [10] can be effectively used for their formation. However, structuring of prepared nanoisland films for the formation of PM is restricted by the fragility of these films, which can result in their degradation under secondary processing. An approach to structuring out-diffused MIF based on thermal poling of ion-exchanged glasses has recently been proposed [11]. In this paper, we present the model of the out-diffused nanoisland formation and MIF growth, respective numerical and experimental results, and demonstrate application of silver out-diffusion combined with thermal poling of a silver ion-exchanged glass for the formation of separated groups of nanoislands that are plasmonic molecules.

## Methods

Processes occurring in glasses enriched with metal ions (further, the metal we consider is silver) under annealing in hydrogen were explored in details elsewhere [4,12]. Oversaturation of the glass with neutral silver formed after hydrogen reduction, generally, results in the formation and growth of both silver nanoparticles in the bulk of the glass and silver nanoislands on the glass surface. The latter is because of bell-shaped depth distribution function of atomic silver [4,12] that initiates a diffusion flux directed towards the surface [12]. Further annealing of the glass in hydrogen results in coagulation and ‘sticking’ of the nanoislands into a solid film.

Thermal poling of glasses leads to the depletion of the subsurface layer with mobile ions, first of all with monovalent ones [13]. In our case, these are silver and sodium ions of the ion-exchanged glass. Generally, thermal processing of the poled glass in hydrogen should provide the reduction of silver ions in the same way as the processing of an unpoled glass; however, there is a difference in the dynamics of the formation of nanoparticles in the bulk and nanoislands on the surface. In particular, the growth of nanoislands and nanoparticles in the glass starts later because silver reduction is delayed by the time necessary for hydrogen to go through the silver-depleted subsurface layer, and the nucleation of silver islands is also delayed by the time necessary for silver atoms to diffuse through the same layer. Thus, poling of an ion-exchanged glass with a structured electrode can result in delayed formation of silver nanoislands on the surface of strongly poled regions of the glass, and proper choice of thermal poling and hydrogen annealing conditions should allow the formation of structured MIF. Moreover, because of silver diffusion towards the glass surface from relatively distant poling-deepen source, the growth of silver nanoislands is slowest at the initial stage of the hydrogen processing. This provides a good possibility for growing well-separated nanoislands and, if the area of the unpoled region of the glass is in nanometer scale, for the formation of small groups of metal nanoislands that are photonic molecules. Moreover, in the case of identical apertures capable of placing a few nanoislands only, formation of very similar structures of nanoislands should be awaited. This similarity is because of diffusion-driven self-arranged process of the nanoisland formation. It is worth to note that the proposed technique allows easy multiplication of formed structures via repeated use of the same electrode [14].

## Results

**Figure 1:**
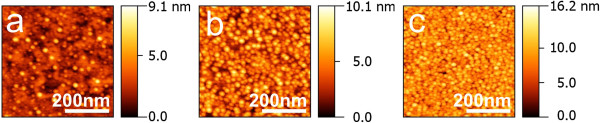
**AFM images of silver nanoislands on the surface of the glass annealed in hydrogen.** AFM images of MIF on the surface of the glass annealed in hydrogen for 10 min **(a)**, 20 min **(b)**, and 330 min **(c)** at 100°C. The glass slides were ion-exchanged in the bath containing 5 wt.% of AgNO_3_ and 95 wt.% of NaNO_3_ at 325°C for 20 min.

In the experiments, we used Menzel [15] microscope slides; the ion-exchanged process is described in details in [11]. The use of ion-exchange bath containing 5 wt.% of AgNO_3_ and 95 wt.% of NaNO_3_ allowed us to replace of approximately 75 wt.% of sodium by silver at the glass surface as followed from our previous studies of silver-sodium ion exchange in soda-lime glasses [16]. Chosen temperature and duration of the ion exchange indicated in Figure 1 caption corresponded to the penetration of silver ions into the glass by 7 μm with the half of maximum concentration at approximately 3.5 μm [16]. Varying hydrogen annealing conditions, we found the mode providing the formation of MIF with clearly separated nanoparticles as shown in Figure 1a,b while essentially longer annealing resulted in the layer of closely packed nanoparticles (Figure 1c). Visually smaller size of the metal islands in Figure 1c is probably because of atomic force microscope tip artifact [17], that is, the decrease of atomic force microscopy (AFM)-measured size for closer placed nanoparticles. This takes place when the average distance between the nanoparticles does not allow the AFM tip to touch substrate everywhere because it is less than the AFM tip edge. Increased thickness of the MIF in Figure 1c also does not indicate the decreased size of the nanoparticles. The images of MIF presented in Figure 1 were obtained using atomic force microscope Veeco Dimension 3100 (AFM; Veeco Instruments Inc., Plainview, NY, USA).

**Figure 2:**
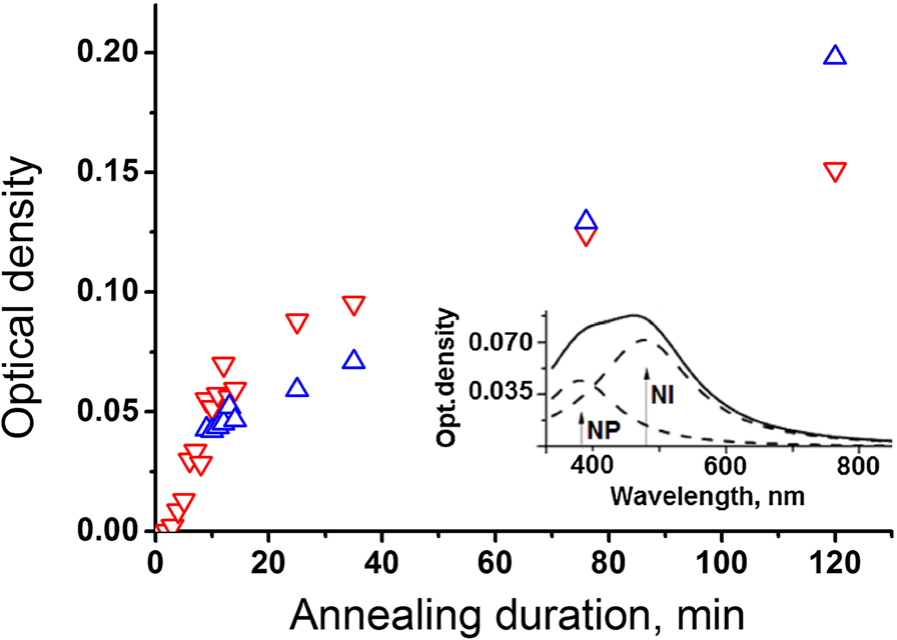
**Input of nanoislands on the surface and nanoparticles in the bulk in SPR absorption.** The optical density of SPR absorption peak due to nanoparticles in the bulk of the glass (triangle) and due to the nanoislands in MIF (inverted triangle). Hydrogen annealing temperature 120°C, ion exchange was performed at 325°C for 20 min. Inset: nanoparticles (NP) and nanoislands (NI) absorption peak in the absorption spectrum of 10-min annealed sample.

To characterize the growth of the MIF, we have measured the optical absorption spectra of the slides. As soon as besides MIF formation on the surface, the formation of nanoparticles in the bulk of the glass takes place and we repeat the spectral measurements after the MIF removal as described in ref. [11] and calculated differential spectra. The peak corresponding to the surface plasmon resonance (SPR) measured at the second step characterized silver nanoparticles formed in the bulk while the SPR peak in the differential spectra characterized silver nanoislands on the glass surface. The dynamics of these peaks growth is illustrated with Figure 2. It is worth to note that the peaks related to nanoislands on the surface and nanoparticles in the bulk absorption were at different wavelengths, shorter for nanoparticles (see inset in Figure 2), and the peaks moved towards longer wavelength with the annealing duration.

**Figure 3:**
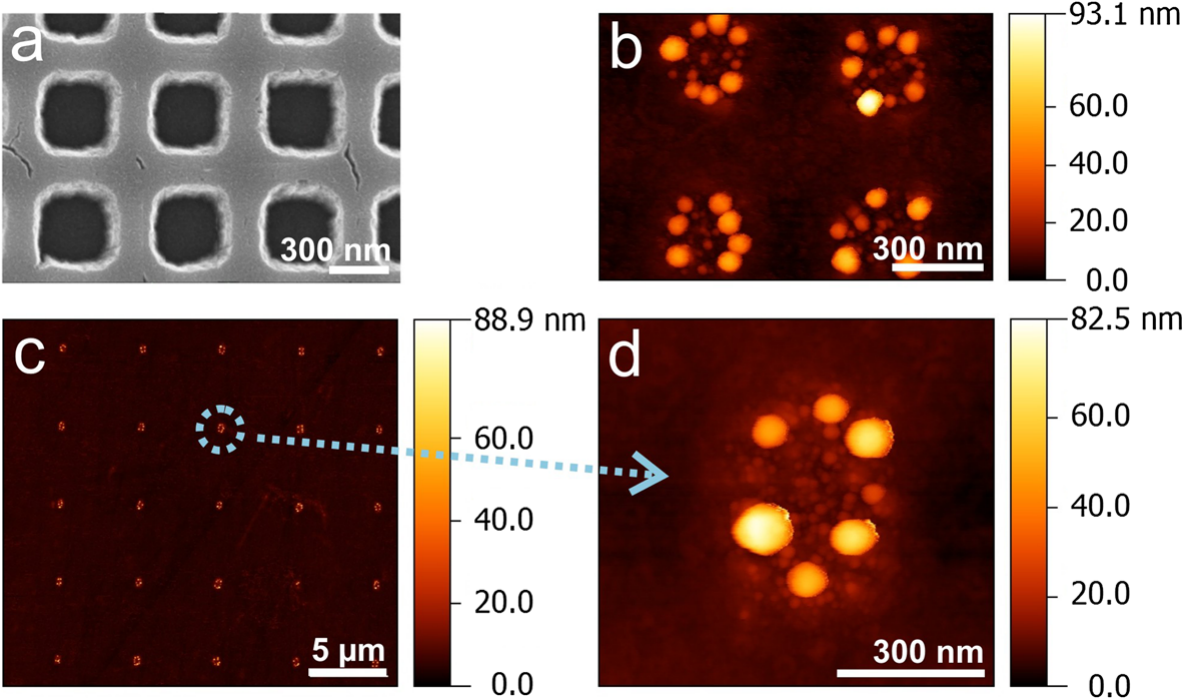
**SEM image of the groups of the silver nanoislands.** SEM image of the part of the electrode (the net of 300 × 300-nm^2^ apertures with 0.6-μm periodicity) used for glass poling **(a)**, AFM images of the groups of silver nanoislands grown after annealing in hydrogen on the surface of the glass poled using this part of electrode **(b)**, and AFM images of the groups of nanoislands grown after poling with another part with 5 μm in period net of 300 × 500-nm^2^ apertures **(c,d)**. The glass slide was ion-exchanged in the bath containing 5 wt.% of AgNO_3_ and 85 wt.% of NaNO_3_ at 325°C for 20 min, poled for 10 s at 300°C using 500 V and, finally, 3 min processed in hydrogen at 315°C.

In the next set of the experiments, we performed thermal poling of the ion-exchanged slides using glassy carbon electrodes, and these poled glasses were annealed in hydrogen. A wide set of poling and hydrogen annealing conditions was tested. According to the results we obtained in modeling the distribution of ions in glasses poled through grating-like aperture [18], the poling time should be minimized to prevent lateral drift of ions resulting in smoothen concentration profiles. Finally, we found a mode allowing the formation of the MIF consisting of well-separated silver nanoislands on the surface of unpoled slides, and, at the same time, there was no formation of the MIF in the poled regions of the glass surface. Then, we poled the ion-exchanged glass slides using the electrode presenting two rectangular nets of 300 × 300 and 300 × 500-nm^2^ apertures with 0.6 and 5-μm periodicity, respectively, made with e-beam lithography followed by ion etching. The depth of the etching was 400 nm. Scanning electron microscope (SEM; Leo 1550 Gemini, Oberkochen, Germany) image of the part of the electrode is presented in Figure 3a. Hydrogen annealing of the poled glass slide has resulted in the set of groups of nanoislands (Figure 3b), mainly containing 6 to 8 silver nanoparticles (Figure 3c,d). Generally, such groups can be considered as plasmonic molecules, which size and number of ‘atoms’ (metal nanoislands).

## Discussion

**Figure 4:**
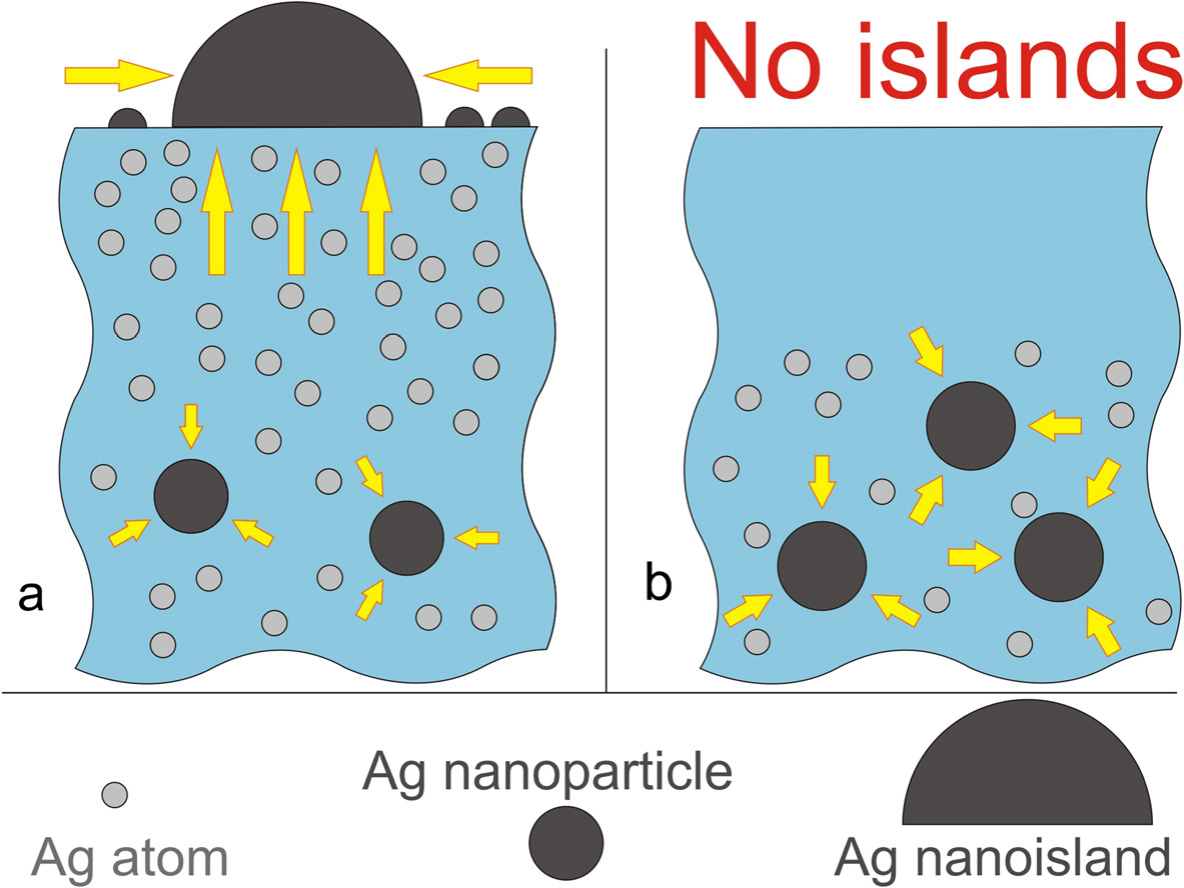
**Mechanisms of silver nanoisland growth.** Surface diffusion of the adatoms and the diffusion of atomic silver from and inside the bulk glass in unpoled **(a)** and poled **(b)** regions.

The mechanism of the MIF formation on the surface of silver ion-exchanged glasses as well as metal nanoparticles in the bulk of the glasses was firstly described in [12]. Later, we modified this theory and accounted for the nanoparticle nucleation dynamics and the dependence of the critical nuclei radii on the oversaturation of the solid solution of silver in the glass matrix [4]. Within the frames of the present study, we have broaden this approach [4] to the growth of nanoislands on the glass surface via introducing surface concentration of silver adatoms and equilibrium surface concentration of silver. Supposing the nanoislands to be hemispherical [6,19] and accounting for the input of silver atoms diffusing both from the bulk of the glass and along the surface (see Figure 4), we constructed the set of equations, which describes the growth of both nanoparticles and nanoislands. Details of the analytical description can be found in the supplementary material.

**Figure 5:**
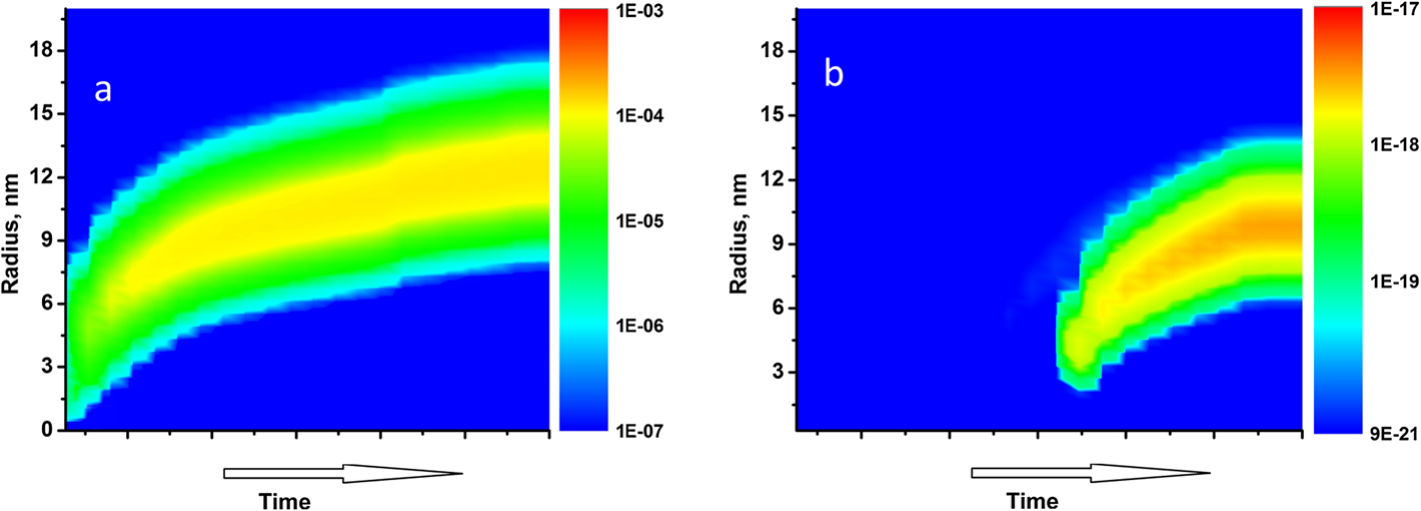
**The temporal evolution of the nanoisland distribution.** The temporal evolution of the nanoisland distribution on the surface of unpoled **(a)** and poled **(b)** ion-exchanged glass. Color indicates the number of islands per nm^2^. Parameters of simulation are taken from [4,12].

Numerical solution of the formulated equations using the finite-difference schemes allowed us to model the dynamics of nanoparticle formation on the glass surface - see Figure 5a. In the modeling, we used data from [16] for temperature dependence of silver ions diffusion coefficient and the approach and numerical data presented in [4,20] to formulate initial conditions corresponding to the case of hydrogen processing of the poled glass. Comparing the dynamics of the nanoisland growth in Figures 2 and 5, one can see the trend to the saturation, that is, the saturation of the effective thickness of the MIF. This saturation is because of preferential consumption of silver atoms by the nanoparticles formed in the bulk of the glass, which absorption grows about linearly for longer annealing of the glass in hydrogen (see Figure 2). Results of the modeling performed for unpoled and poled glass illustrate the time gap between formations of MIF in these two cases. Moreover, poling-induced depletion of the subsurface layer of the glass with silver ions essentially decreases both concentration and average size of the nanoislands grown on the poled surface of the glass. This is because of the distance of silver ions - hydrogen reaction frontier from the glass surface, which results in slow nucleation on the surface and, respectively, preferential growth of nanoparticles in the vicinity of the frontier that is in the bulk of the glass. At the beginning of the hydrogen annealing of the unpoled glasses that interaction frontier is close to the surface that allows fast nucleation of the nanoislands which, when arise, become a strong sink for neutral silver, the strength of this sink decreases with the move of the interaction frontier from the glass surface.Comparing the calculations performed and AFM images of the nanoparticles, one can notice that the measured size of the nanoislands exceeds the calculated one. We relate this to the absence of the flow of silver atoms to the growing nanoparticles from the glass surface out of the unpoled aperture as there are no silver atoms on the surface of the poled glass. This decreases surface oversaturation and can even result in Ostwald repining of the nanoislands that is to the formation of bigger islands which are seen in Figure 3. It is worth to note that the similarity of the formed groups of the nanoislands is because of their self-arranged growth within the area limited by the aperture used in the glass poling.

## Conclusions

Finally, we have demonstrated the growth of a net of small groups of silver nanoislands on the surface of poled silver ion-exchanged glass having the set of unpoled regions 300 × 300 nm^2^. Annealing of the poled glass slide in hydrogen resulted in the formation of the set of similar in geometry plasmonic molecules consisted of 6 to 8 silver nanoislands each. Performed modeling, which accounts for the nanoislands, grows via two mechanisms: surface diffusion and diffusion from the bulk, explains the processes taking place in the formation of these structures. Proposed technique allows multiplication (imprinting) of the sets of plasmonic molecules using the same electrode that provides easy formation which does not require electron beam lithography. We believe that the varying of the size and shape of the apertures could allow formation of the plasmonic molecules differing in number of the nanoislands, their size, and mutual position.

## Abbreviations

AFM: atomic force microscopy; MIF: metal island film; PM: plasmonic molecules; SEM: scanning electron microscopy; SERS: surface-enhanced Raman scattering; SPR: surface plasmon resonance.

## Competing interests

The authors declare that they have no competing interests.

## Authors’ contributions

AR developed the model and performed the numerical calculations. SC made the e-beam lithography and performed the SEM measurements. AB performed the AFM studies of the samples. IR prepared the nanoisland film samples and measured the absorption spectra. VZh critically analyzed the results, and AL supervised the whole work. All authors read and approved the final manuscript.

## References

[B1] Jimenez J Lysenko S Liu H Photoluminescence via plasmon resonance energy transfer in silver nanocomposite glasses J Appl Phys 2008 104 054313 10.1063/1.2976171

[B2] Lipovskii A Melekhin V Petrov M Svirko Y Zhurikhina V Bleaching versus poling: comparison of electric field induced phenomena in glasses and glass-metal nanocomposites Appl Phys Rev 2011 109 011101 10.1063/1.3511746

[B3] Quaranta A Cattaruzza E Gonella F Rahman A Mariotto G Cross-sectional Raman micro-spectroscopy study of silver nanoparticles in soda-lime glasses J Non-Cryst Solids 2014 401 219

[B4] Redkov A Zhurikhina V Lipovskii A Formation and self-arrangement of silver nanoparticles in glass via annealing in hydrogen J Non-Cryst Sol 2013 376 152 157

[B5] De Marchi G Caccavale F Gonella F Mattei G Mazzoldi P Battaglin G Quaranta A Silver nanoclusters formation in ion-exchanged waveguides by annealing in hydrogen atmosphere Appl Phys A 1996 63 403 407

[B6] Zhurikhina VV Brunkov PN Melehin VG Kaplas T Svirko Y Rutckaia VV Lipovskii AA Formation of metal island films for SERS by reactive diffusion Nanoscale Res Lett 2012 7 676 10.1186/1556-276X-7-676 23244007 PMC3563545

[B7] Bolesta I Kushnir O Kolych I Syvorotka I Adamiv V Burak Y Teslyuk I AFM investigations and plasmon spectra of silver clusters formed on Li_2_B_4_O7:Ag glass surface in reducing atmosphere Advanced Sci Eng Med 2014 6 1 7 10.1166/asem.2014.1442

[B8] Chervinskii S Matikainen A Dergachev A Lipovskii A Honkanen S Out-diffused silver island films for surface-enhanced Raman scattering protected with TiO_2_ films using atomic layer deposition Nanoscale Res Lett 2014 9 398 10.1186/1556-276X-9-398 25170333 PMC4141881

[B9] Wang H Brandl DW Nordlander P Halas NJ Plasmonic nanostructures: artificial molecules Acc Chem Res 2007 40 53 10.1021/ar0401045 17226945

[B10] Tan SJ Campolongo MJ Luo D Cheng W Building plasmonic nanostructures with DNA Nat Nanotechnol 2011 6 268 276 10.1038/nnano.2011.49 21499251

[B11] Chervinskii S Sevriuk V Reduto I Lipovskii A Formation and 2D-patterning of silver nanoisland film using thermal poling and out-diffusion from glass J Appl Phys 2013 114 224301 10.1063/1.4840996

[B12] Kaganovskii Y Lipovskii A Rosenbluh M Zhurikhina V Formation of nanoclusters through silver reduction in glasses: the model J Non-Cryst Solids 2007 353 2263 2271 10.1016/j.jnoncrysol.2007.03.003

[B13] Lepienski CM Giacometti JA Ferreira CFLG Freire FL Achete CA Electric field distribution and near-surface modifications in soda-lime glass submitted to a DC potential J Non-Cryst Solids 1993 159 204 212 10.1016/0022-3093(93)90224-L

[B14] Lipovskii AA Melehin VG Petrov MI Svirko YP Hennessy TC Thermal electric field imprinting lithography: fundamentals and applications, edited collection “Lithography: Principles, Processes and Materials” 2011 6 400 Oser Ave Suite 1600, Hauppauge NY 11788-3619, USA: Nova Science Publishers, Inc 149 163

[B15] Menzel-Glaser: microscope slides Menzel-Glaser: microscope slides http://www.menzel.de/Microscope-Slides.687.0.html?&L=1

[B16] Zhurikhina VV Petrov MI Sokolov KS Shustova OV Ion exchange characteristics of sodium-calcium-silicate glass: calculation from mode spectra Tech Phys 2010 55 1447 1452 10.1134/S1063784210100087

[B17] Shiramine K Muto S Shibayama T Sakaguchi N Ichinose H Kozaki T Sato S Nakata Y Yokoyama N Taniwaki M Tip artifact in atomic force microscopy observations of InAs quantum dots grown in Stranski-Krastanow mode J Appl Phys 2007 101 033527 10.1063/1.2434806

[B18] Sokolov K Melehin V Petrov M Zhurikhina V Lipovskii A On spatially periodical poling of silica glass J Appl Phys 2012 111 104307 10.1063/1.4714350

[B19] Obraztsov P Nashchekin A Nikonorov N Sidorov A Panfilova A Brunkov P Formation of silver nanoparticles on the silicate glass surface after ion exchange Phys Solid State 2013 55 1272 1278 10.1134/S1063783413060267

[B20] Lipovskii AA Omel’chenko AA Petrov MI Modeling charge transfer dynamics and electric field distribution in glasses during poling and electrostimulated diffusion Tech Phys Lett 2010 35 1028 1031

