# Erratum to: Plasmonic molecules via glass annealing in hydrogen

**DOI:** 10.1186/s11671-015-0867-6

**Published:** 2015-04-28

**Authors:** Alexey Redkov, Semen Chervinskii, Alexander Baklanov, Igor Reduto, Valentina Zhurikhina, Andrey Lipovskii

**Affiliations:** Institute of Physics, Nanotechnology and Telecommunications, St. Petersburg State Polytechnic University, 29 Polytechnicheskaya, St. Petersburg, 195251 Russia; Department of Physics and Technology of Nanostructures, St. Petersburg Academic University, 8/3 Khlopina, St. Petersburg, 194021 Russia; Institute of Photonics, University of Eastern Finland, Yliopistokatu 7, P.O. Box 111, Joensuu, 80101 Finland; Ioffe Physical-Technical Institute of RAS, 26 Polytechnicheskaya, St. Petersburg, 194021 Russia

## Erratum

The original version of this article [1] referred to supplementary material which was subsequently not made available alongside the publication. The material can be found as part of this erratum (Additional file 1). The publisher would like to apologise to the authors for this oversight.

## Additional file

Additional file 1: **Plasmonic molecules via glass annealing in hydrogen: supplementary materials.**
