# Supplementary material for: Erratum to: Plasmonic molecules via glass annealing in hydrogen
Source: Nanoscale Res Lett. 2015 Apr 28;10:201. doi: 10.1186/s11671-015-0867-6 (PMC4411328; doi:10.1186/s11671-015-0867-6)

**Plasmonic molecules via glass annealing in hydrogen**

Alexey Redkov ^1,2^

Email:[avredkov@gmail.com](mailto:red-alex@mail.ru)

Semen Chervinskii ^3,1,*^
Email: [semen.chervinskii@uef.fi](mailto:semen.chervinskii@uef.fi)

Alexander Baklanov ^1,4^

Email:[baklanov@mail.ioffe.ru](mailto:baklanov@mail.ioffe.ru)

Igor Reduto ^2^

Email:[reduto-igor@rambler.ru](mailto:reduto-igor@rambler.ru)

Valentina Zhurikhina ^1^

Email: [zhurikhina@spbau.ru](mailto:zhurikhina@spbau.ru)

Andrey Lipovskii ^1,2^

Email: [lipovsky@spbau.ru](mailto:lipovsky@spbau.ru)

^1^Institute of Physics, Nanotechnology and Telecommunications, St. Petersburg State Polytechnic University, 29 Polytechnicheskaya, St. Petersburg 195251, Russia

^2^Department of Physics and Technology of Nanostructures, St. Petersburg Academic University, 8/3 Khlopina, St. Petersburg 194021, Russia

^3^Institute of Photonics, University of Eastern Finland, P.O. Box 111, Joensuu FI-80101, Finland

^4^Ioffe Physical-Technical Institute of the RAS, 26 Polytekhnicheskaya, St. Petersburg 194021, Russia

^*^ Corresponding author. Institute of Photonics, University of Eastern Finland, P.O. Box 111, Joensuu FI-80101, Finland

**Supplementary materials**

**The model of metal island film formation**

As shown in [4,12], there are a few entities, participating in this process, namely, silver ions (Ag^+^), hydrogen (H^+^) and sodium (Na^+^) (Na^+^ ions do not participate in the reduction reaction, but affect the diffusion of the silver ions and hydrogen), atoms of silver (Ag^0^) and hydrogen (H^0^), as well as islands at the surface and nanoparticles in the bulk. Since the size of the glass sample essentially exceeds characteristic diffusion lengths of all the reagents, we further consider a one-dimensional problem of the diffusion of hydrogen from the surface of the glass into the half-space. The equations for the description of the volume concentration of silver ions and sodium, atomic silver and the formation and growth of nanoparticles were formulated in [4], so hereinafter, we will use them and notation adopted in that work.

Let us consider the formation and growth of hemispherical islands on the surface. By analogy with the growth in the bulk glass, we introduce surface concentration of silver adatoms and silver surface solubility or equilibrium concentration (superscript «s» means surface). We also assume that they are proportional to , (see [4]), respectively (in general this may not be the case). Surface supersaturation in this case would be defined as .

Then, the critical radius of the hemispherical island can be written as , and the number of nuclei generated per time unit:

(1)

Here and are analogues of three-dimensional coefficients and , introduced in [4]. Then one can write the following equation of continuity for distribution function of islands (*R,t*), (similar to equation for nanoparticles in the bulk).

(2)

Now let’s find the growth rate of an island of radius *R*: *V^S^* (*R*). There are two possible growth mechanisms: diffusion of atoms of neutral silver from bulk glass and joining of the adatoms of silver, which are diffusing on the surface of the glass, as illustrated in Figure 4. The number of atoms per area unit absorbed by the lower surface of the island of radius *R* per time unit can be calculated as follows:

(3)

The number of atoms that are absorbed via the surface mechanism is described by the formula:

(4)

It should be noted that the expression (4) is correct if the distances between the islands are much larger than their typical size. Only in this case one can suppose that concentration field, which is assumed in the derivation of this formula, have been established. However, it will be shown later that during the growth of the islands, one mechanism replaces another, and when the islands are large enough, the main mechanism of growth is growth via diffusion from the bulk glass.

Volume of nanoparticle increases with the rate $\omega\left(J_{1}(R)+J_{2}(R)\right)$, and the growth rate of the radius can be written as:

(R)= $\frac{dR}{dt}$= (5)

Thus, total consumption of atomic silver on the growth of the particles of radius R:

=+

To calculate the total flow it is necessary to integrate $J(R)$ and take into account atoms that participate in the formation of new islands:

. (6)

We suppose that the system at any time is in quasi-equilibrium, i.e., the number of silver atoms are absorbed on the border is equal to the quantity of the atoms diffused from the bulk of the glass:

(7)

Hence we find the boundary condition for the equation for neutral silver from [4]:

(8)

The expression in the denominator imposes a natural restriction of the model: the proportion of the area occupied by the islands must be less than unity.

Thus, we modified the model [4] via addition of the equation for the formation and growth of islands on the surface of the glass, and changing the boundary condition for the bulk concentration of atomic silver in accordance with (8). A system of equations was solved by the finite- difference schemes method.

**Parameters used for modeling**

Parameters (diffusion coefficients, surface tension, etc.) used in the simulation were taken from various sources [4, 8]. The parameters were varied within a wide range for the analysis of the possible results of the process and to compare with known experimental data. Parameters and ranges of their variation are presented in Table 1.

**Table 1**– Parameters used in modeling simulation.

| ,  nm^2^/sec | nm | $C_{1}$, nm^-3^ | $C_{0}$, nm^-3^ |
| --- | --- | --- | --- |
| 10-10^2^ | 0.5-2 | 0.01-1 | 0.1-5 |

**Results of the modeling**

For depths exceeding the width of the distribution of the concentration of neutral silver, all the results relating to the bulk nucleation obtained without the formation of islands [4] remain correct. On the other hand, we can assume that by the time when front of the reactive diffusion reaches such depths, the process of formation and growth of islands is finished. Let us consider the evolution of distributions of the reactants involved in this process in the interval of time corresponding to formation of island film.

*Distribution of hydrogen and silver*

The depth distribution functions for hydrogen and ionic silver have not been changed with respect to [4], because the concentration of atomic silver have no impact on hydrogen and silver ions, whereas we have modified only equation for atomic silver. The latter has been slightly changed (Fig. S1), because now it has different boundary condition and non-zero flux at the surface.


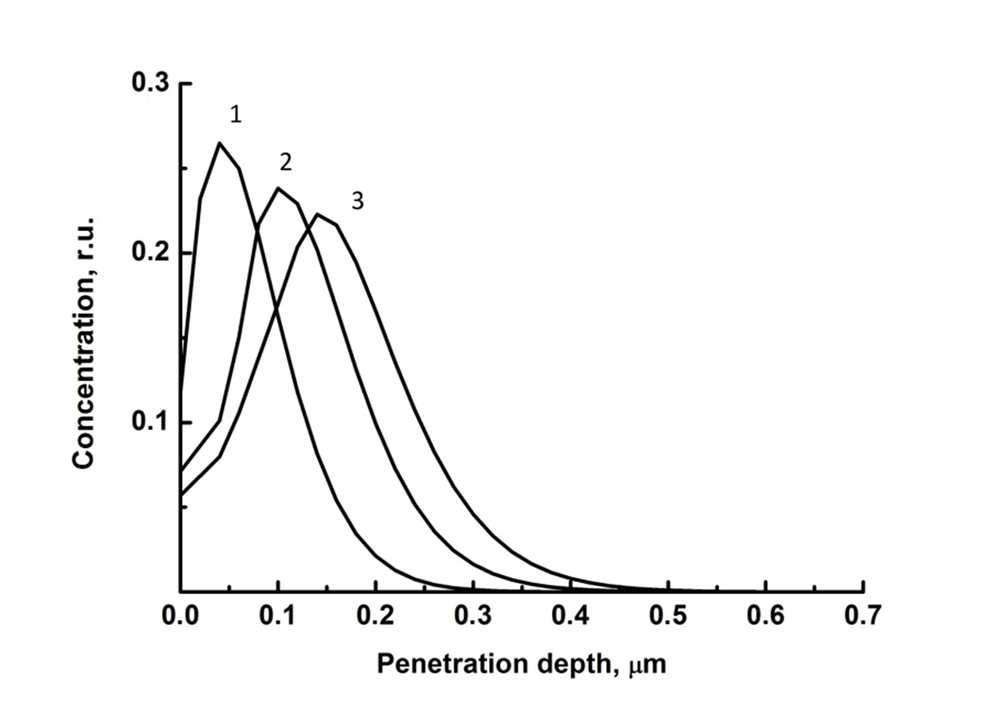


Figure S1. Depth distribution of atomic silver at different times: 2 min (1), 4 min (2), 6 min (3). The normalization is performed on the initial concentration of silver ions.

The concentration of atomic silver is bell-shaped and from a certain point of time, the concentration gradient becomes almost zero at the surface. At this point, the growth of islands stops and atomic silver is absorbed mostly by the formation and growth of nanoparticles in the bulk. More details of this process are discussed in [4].

*Formation and growth of MIF*

Let us consider the dynamics of the growth of metal islands (Figure 3). As was mentioned before, the film forming process ends in the first minutes (tens of minutes) after the beginning of the annealing. During this time the bulk glass accumulates a sufficient amount of atomic silver, and the stage of formation and growth of nanoparticles in the bulk begins, as described in [4]. Nanoparticles are a strong sink for atomic silver, so the flow of silver to the surface weakens and the growth of islands stops. We have calculated the effective thickness of MIF - thickness of uniformly deposited on the surface metallic layer, which is equal in volume to the sum of volumes of all islands. Because is the number of islands per area unit, can be expressed as follows:

(9)

Figure 4 shows the dependence of the average thickness of the film on the various parameters of annealing.


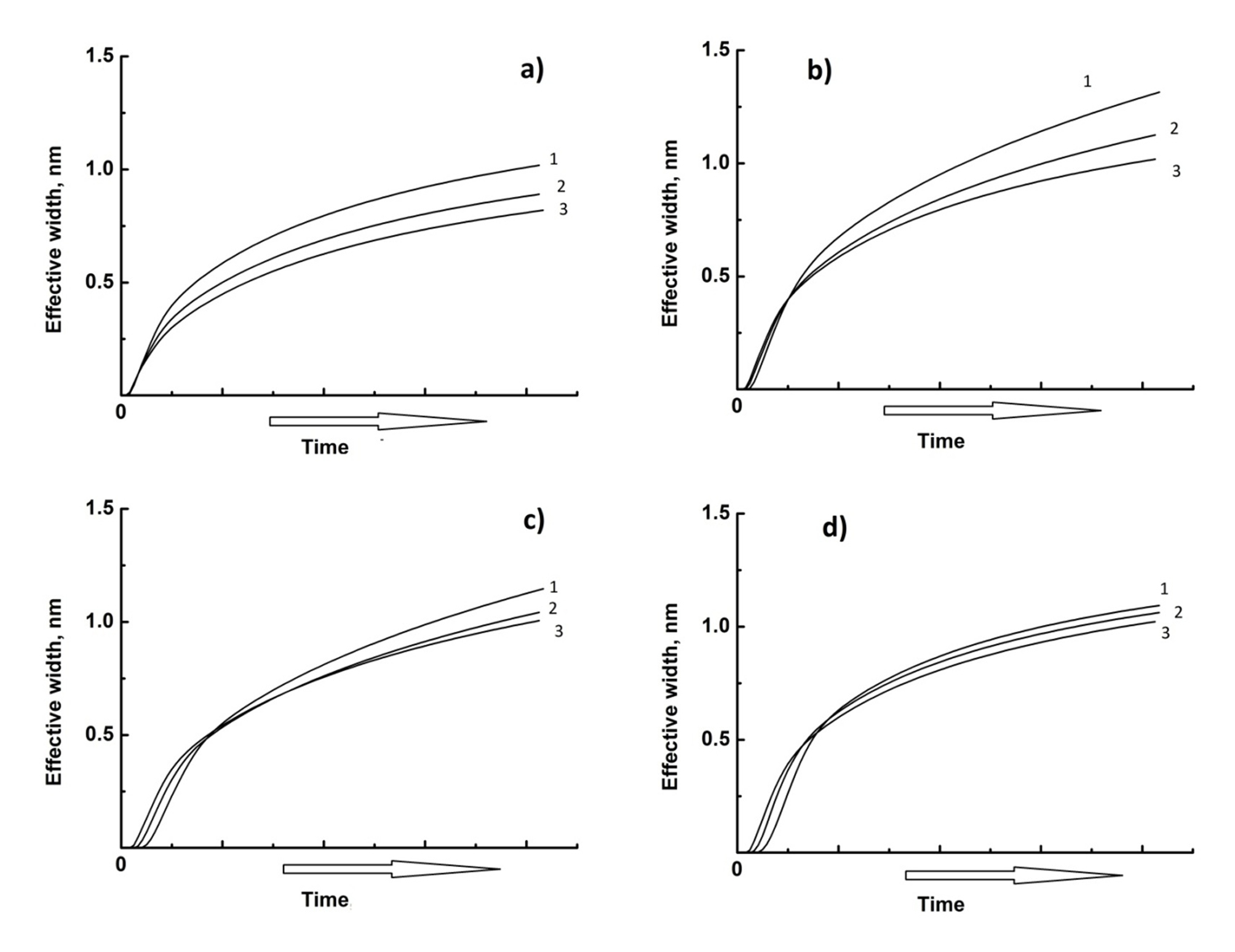


Figure S2. The dependence of the effective thickness of the film on the coefficient of diffusion of atomic silver:$D_{Ag0}=30$(1) , $D_{Ag0}=20$ (2), nm^2^/sec (3) - (a); on the hydrogen diffusion coefficient: $D_{H0}=15$(1) $D_{H0}=$75 (2),$D_{H0}=3\cdot10^{2}$ nm^2^/sec (3) - (b); on the initial concentration of hydrogen near the glass surface: $C_{H0}=0.15$ (1), $C_{H0}=0.25$ (2), $C_{H0}=0.4$ atoms/nm^3^ (3) - (c); on the initial concentration of silver ions in glass:$C_{Ag0}=0.8$ (1), $C_{Ag0}=1.3$ (2), $C_{Ag0}=2$ atoms/nm^3^ (3) - (d).

**Discussion: the influence of the system parameters**

As it can be seen from the simulation results, the distribution function of islands depends on several parameters: the initial concentration of hydrogen ions and silver, diffusion coefficients (temperature), the coefficients $k_{1}^{^{s}}$ and $k_{2}^{^{s}}$, and others. Processes occurring in the bulk glass were analyzed in [4], so we will not consider the parameters that determine the growth of nanoparticles ($k_{1}$,$k_{2}$,$k_{3}$), and will discuss only the results presented above.

*The diffusion coefficient of atomic silver*

Coefficient almost has no effect on the effective thickness of the film at the initial stage of the growth of islands (Figure 4a), since they grow primarily by the mechanism of surface diffusion (surface supersaturation still large and islands radii are small). When the islands grow larger, the mechanism of growth due to the influx of silver from the bulk begins to dominate. Therefore, reducing of this coefficient at this stage of growth leads to decrease in growth rate of islands.

*The diffusion coefficient of hydrogen*

As it follows from the results of numerical calculations (Figure 4b), the higher the hydrogen diffusion coefficient, the thinner the resulting film. This can be explained by the fact that an increase in the diffusion of hydrogen leads to earlier oversaturation of solid solution of atomic silver in the glass, and the active formation and growth of nanoparticles in the bulk glass begins. Nanoparticles become a powerful sink for silver atoms diffusing towards the surface. Therefore the amount of silver, which has reached the surface, becomes less and less.

*The initial concentration of silver ions and hydrogen*

The amount of silver contained in the glass at the beginning of the annealing process considerably affects both the concentration of nanoparticles in the bulk and the final thickness of the film. Increase in initial concentration of silver leads, firstly, to increase in the flow of atomic silver towards the surface and, secondly, to the slower hydrogen diffusion because an increasing part of the hydrogen reacts with silver ions.

In our experimental conditions concentration $C_{H0}$ is less than for an order of magnitude or comparable with it, so the qualitative effect of their change is approximately the same. Decrease in $C_{Ag0}$ and $C_{H0}$ leads to slower formation of atomic silver, it takes longer to reach the critical value of supersaturation, and the beginning of formation of island film starts later. Also one can note, that lower (but still enough to activate film growth) initial concentrations $C_{Ag0}$ and $C_{H0}$ result in thicker film. It can be explained by the fact, that maximal supersaturation in the bulk also becomes lower, bulk nucleation is suppressed, so more atomic silver is being absorbed by the surface.


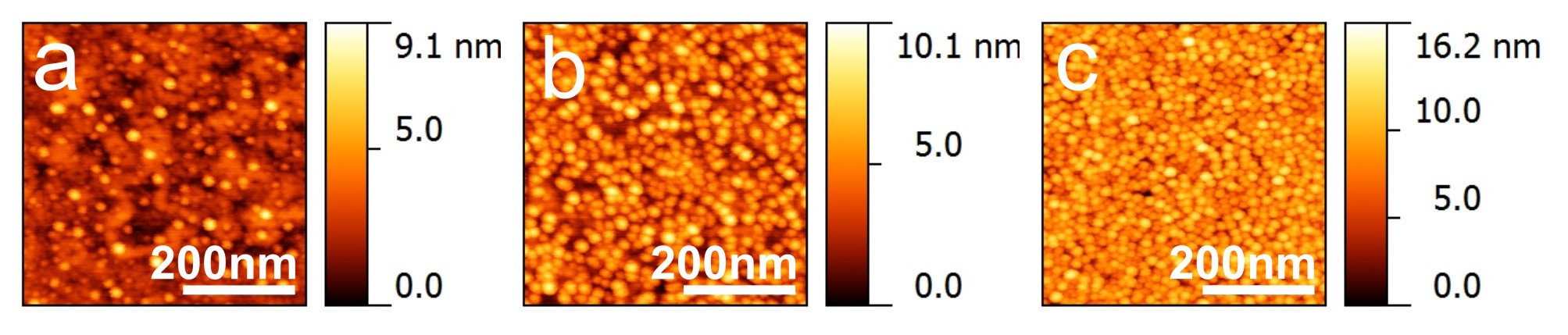


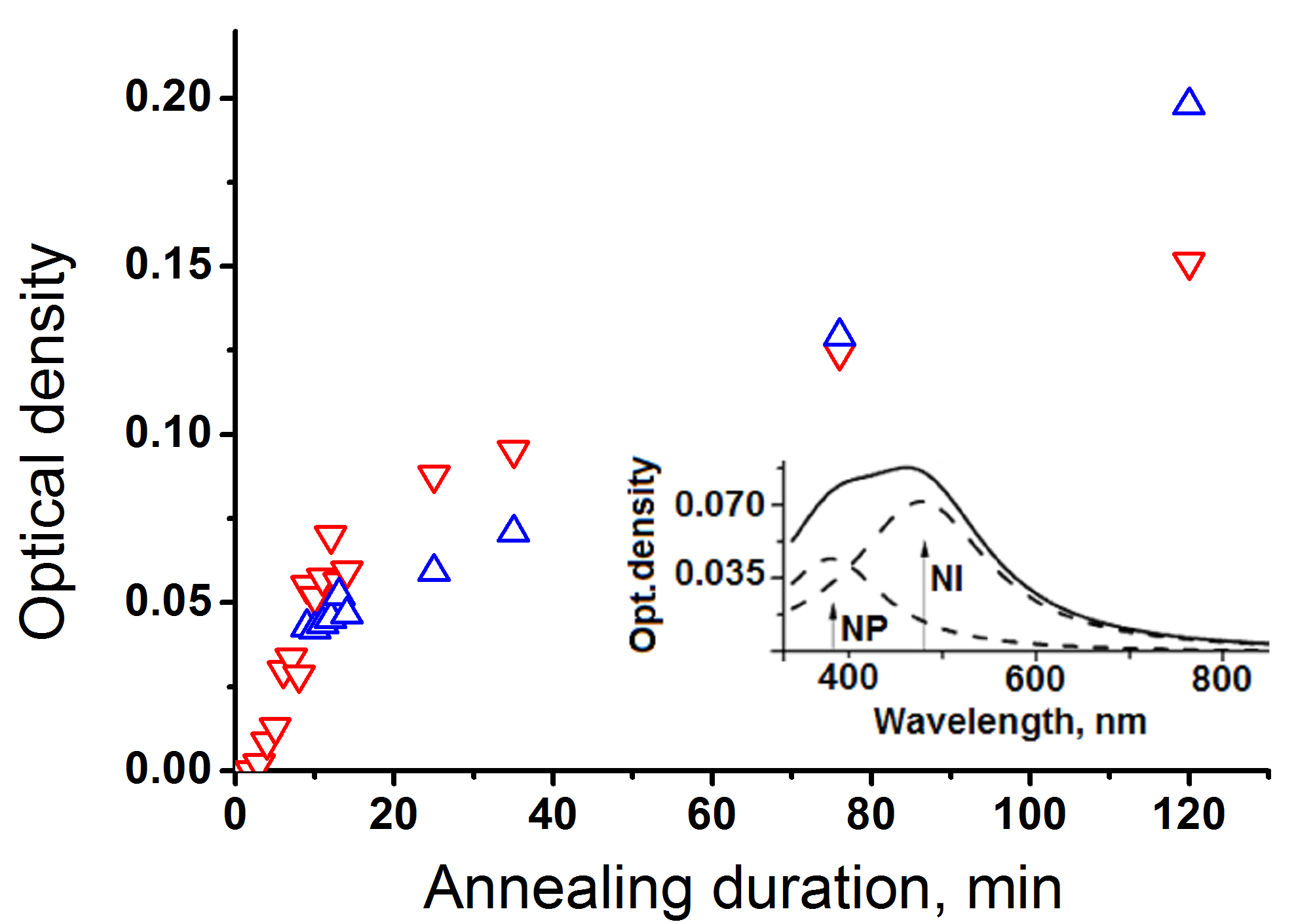


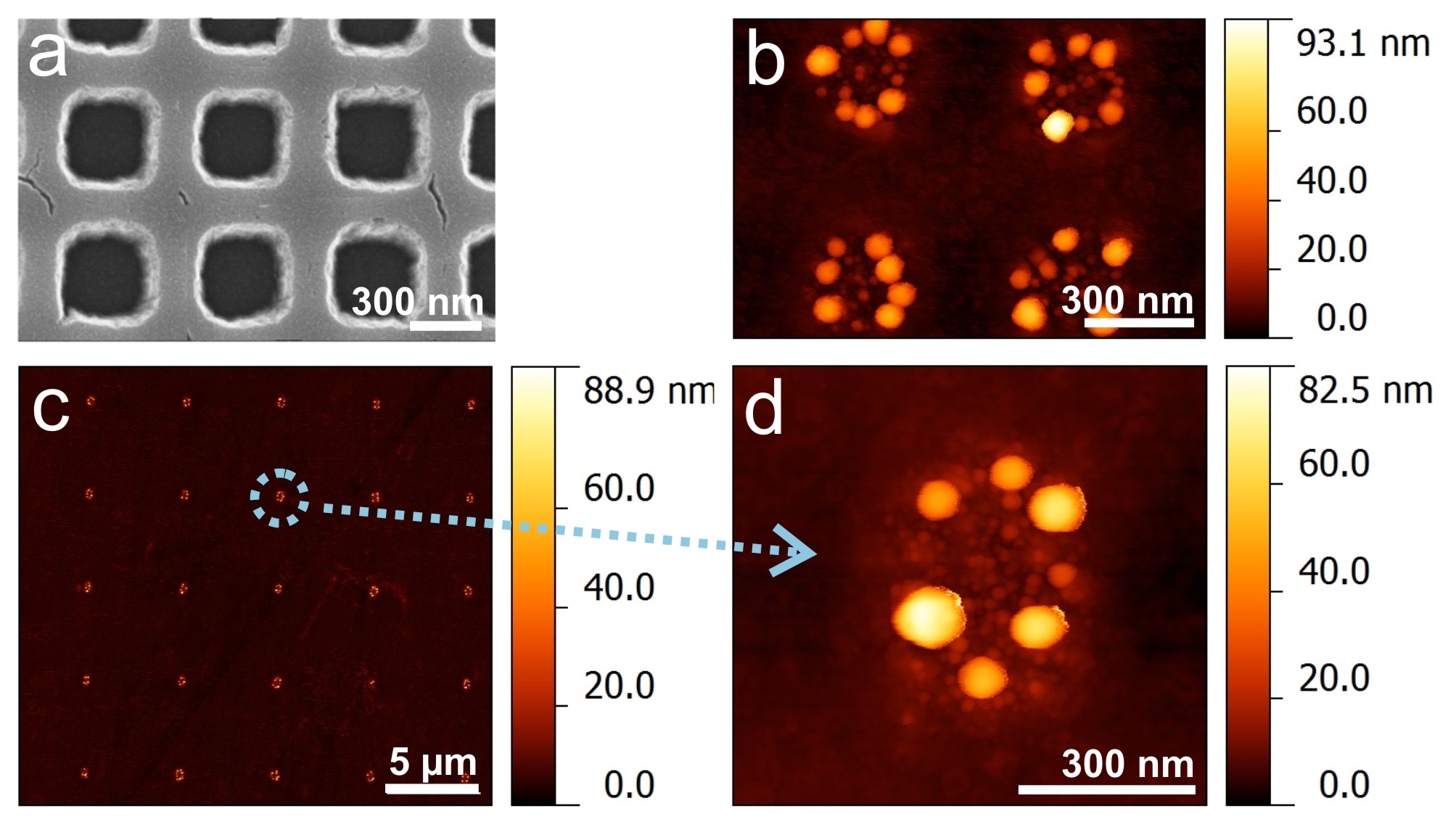


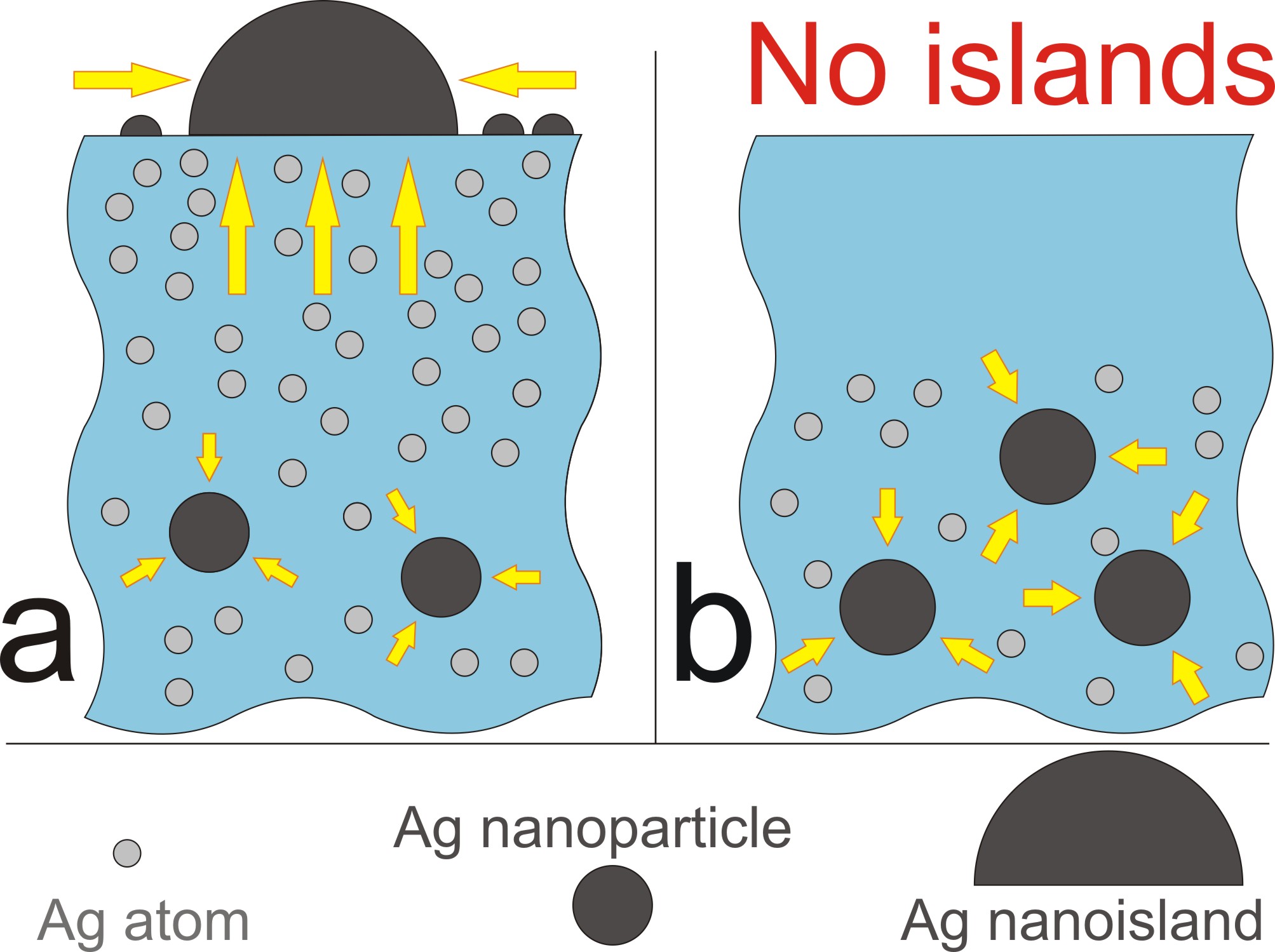


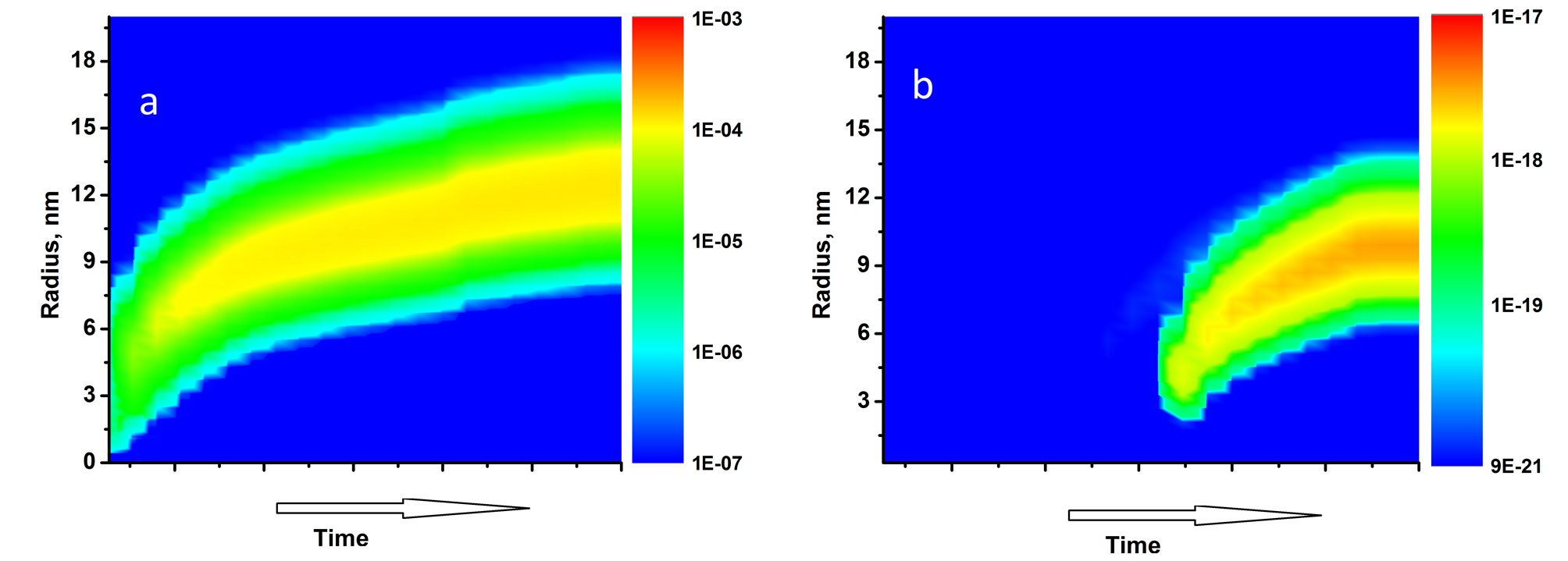

Supplement: Supplementary file 1 — Plasmonic molecules via glass annealing in hydrogen: supplementary materials. [file 11671_2015_867_MOESM1_ESM.docx]
